# Supplementary material for: Simultaneous sensing and imaging of individual biomolecular complexes enabled by modular DNA–protein coupling
Source: Commun Chem. 2020 Feb 12;3:20. doi: 10.1038/s42004-020-0267-4 (PMC9814868; doi:10.1038/s42004-020-0267-4)
Supplement: Supplementary file 1 — Supplementary Information [file 42004_2020_267_MOESM1_ESM.pdf]

Supplementary information

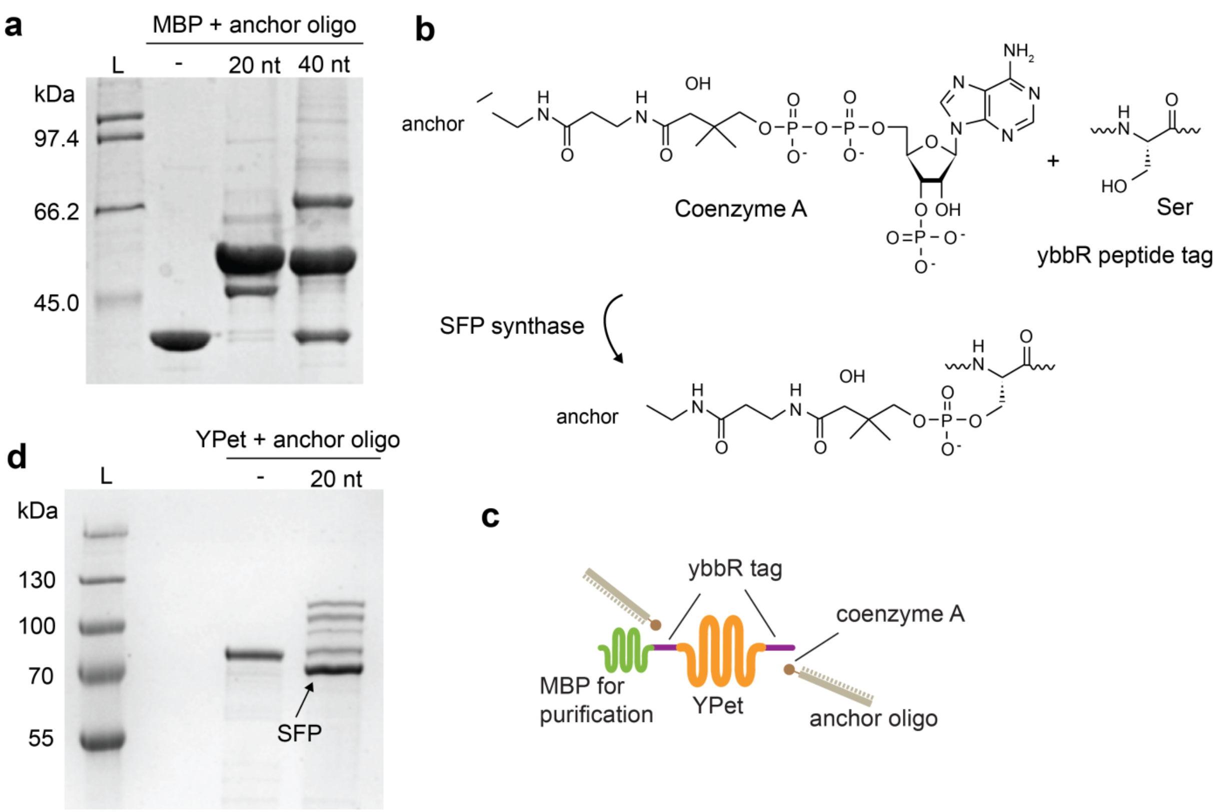

Supplementary Fig. 1: Additional coupling of anchors to proteins. **a**, SDS-PAGE showing the coupling reaction for MBP using 20 and 40 nt anchors. Coupling yield quantification is shown in Figure 1d. **b**, SFP-catalyzed reaction of CoA and the ybbR peptide (adapted from Zhou et al.)[1]. **c**, Scheme of the coupling using the SFP synthase approach for proteins with essential cysteines (YPet here). SFP synthase transfers the 4'-phosphopantetheinyl moiety of CoA to a serine residue of the ybbR tag. **d**, SDS-PAGE showing YPet (lane 1) coupling to a 20 nt anchor modified with coenzyme A (lane 2). SFP enzyme band is indicated with an arrow and lies below uncoupled YPet. Double anchor coupling yield is 27%. There are two bands corresponding to single-coupling due to the position of the tags, one at the C-terminus of YPet and the other one between MBP and YPet, giving rise to different migration behaviors.

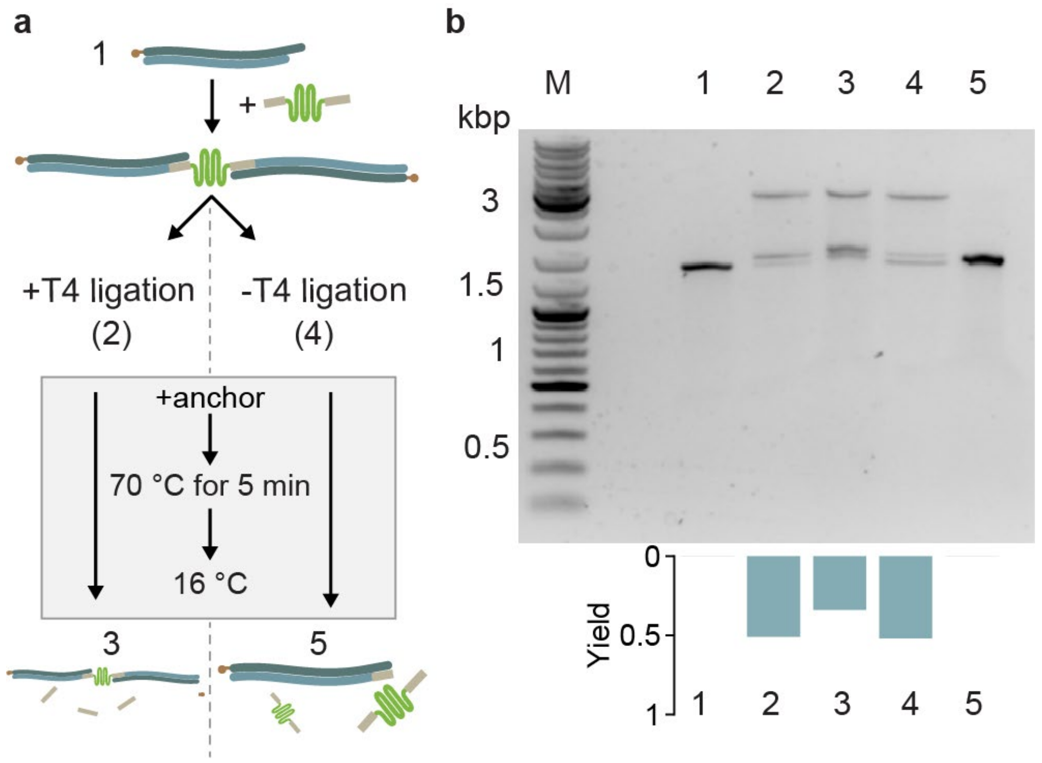

Supplementary Fig. 2: success of the ligation reaction prevents construct disassembly upon heat treatment. a, The ligated and non-ligated constructs were set to 70 °C in the presence of 100x excess free anchor for 5 minutes and subsequent cooling down to 16 °C. **b**, Lane 1: 1300 bp DNA handle with overhang alone. Lane 2: Ligated construct (top band is 49% of total product). Lane 3: ligated construct after the heat treatment. A large portion of the construct remained intact (75% of total, in agreement with the fraction of tethers that can reach overstretching, see Fig. 3). Lane 4: non-ligated construct (top band is 47% of total product). Lane 5: non-ligated construct after heat treatment. The high temperature melted the short hybridized segments and the free anchor replaced the protein, thus dismantling the two-handle attachments completely.

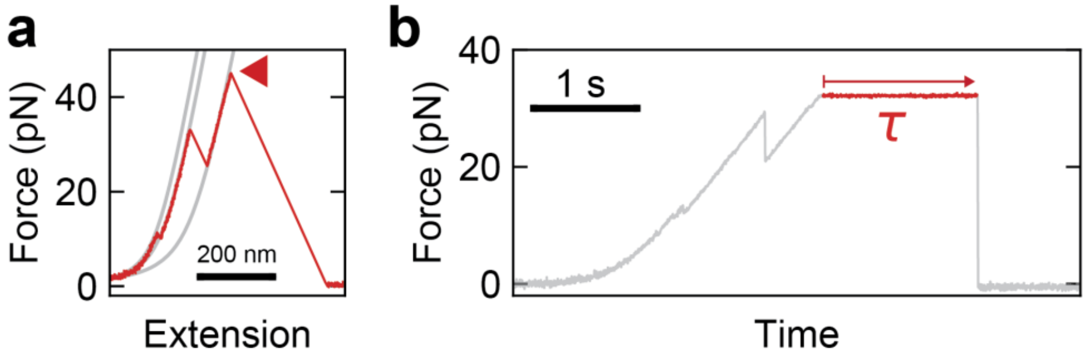

Supplementary Fig. 3: Tether resistance quantifications. a, characterization of the maximum sustained force for a breaking tether (red triangle). Gray lines indicate WLC fits. **b**, example of tether lifetime (τ) measurement at 30 pN. For both quantifications, only tethers that display the typical MBP unfolding pattern are considered.

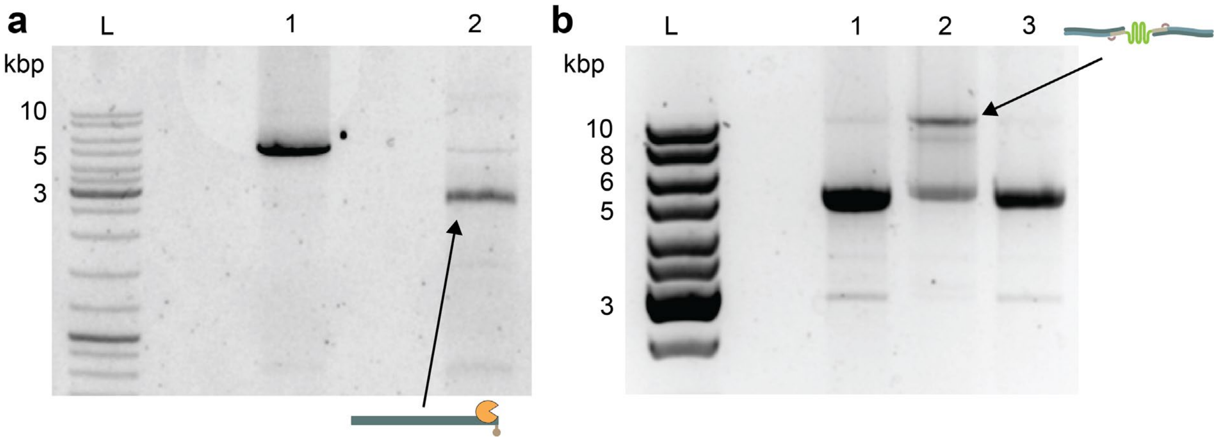

Supplementary Fig. 4: Tethering with 5000 bp DNA handles. a, First amplification of the 5000 bp template (lane 1) and exonuclease digestion (lane 2). **b,** Lane 1 shows the final handle with the overhang after Deep Vent (exo-) PCR. In lane 2 the ligation of the handles with anchor-MBP-anchor shows a clear band at 10 kbp, indicating that the coupling is successful (35%). Lane 3 shows ligation in the absence of anchored protein, with no additional band at 10 kpb indicating that unspecific ligation is negligible.

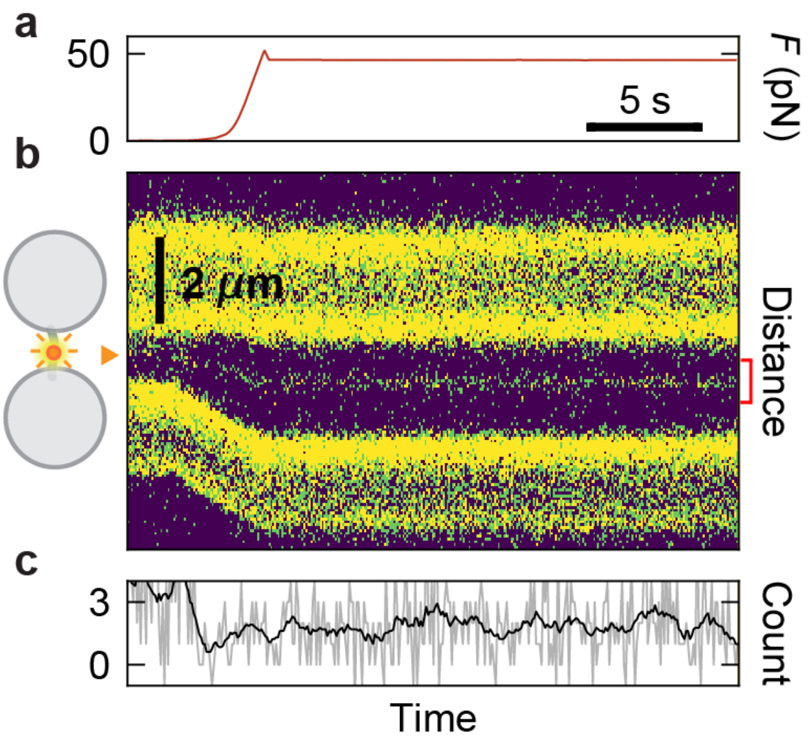

Supplementary Fig. 5: Long tethers (5 kbp) can also sustain high forces for prolonged periods of time while monitoring fluorescence. a, Force monitoring of YPet using optical tweezers. **b**, Confocal scanning kymograph showing active Ypet between the beads. **c**, Photon count from YPet location (region indicated in red in b), after background subtraction.

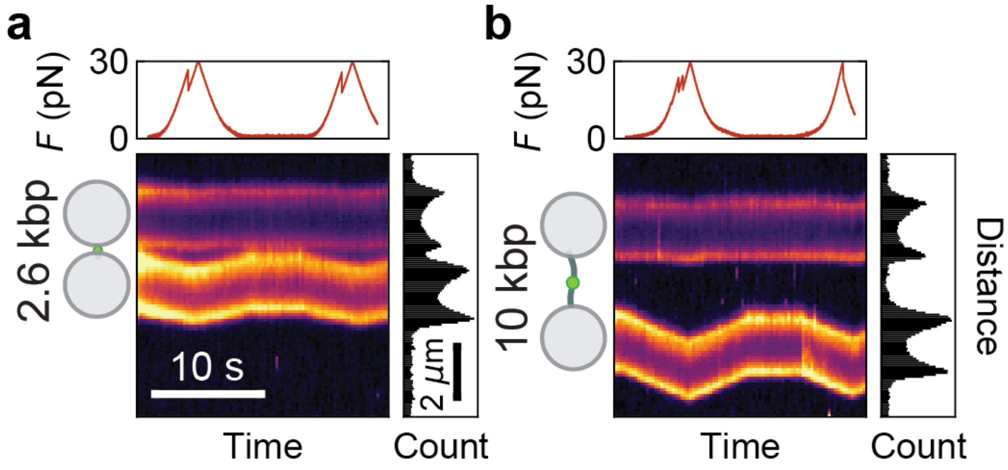

Supplementary Fig. 6: Confocal fluorescence kymographs of MBP in the presence of trigger factor. **a**, 1.3 kbp handles do not provide enough spacing between the beads and noise hinders binding detection, while **b**, 5 kbp handles solve this limitation. The monitored force is shown on top and a typical scanning line profile on the right.

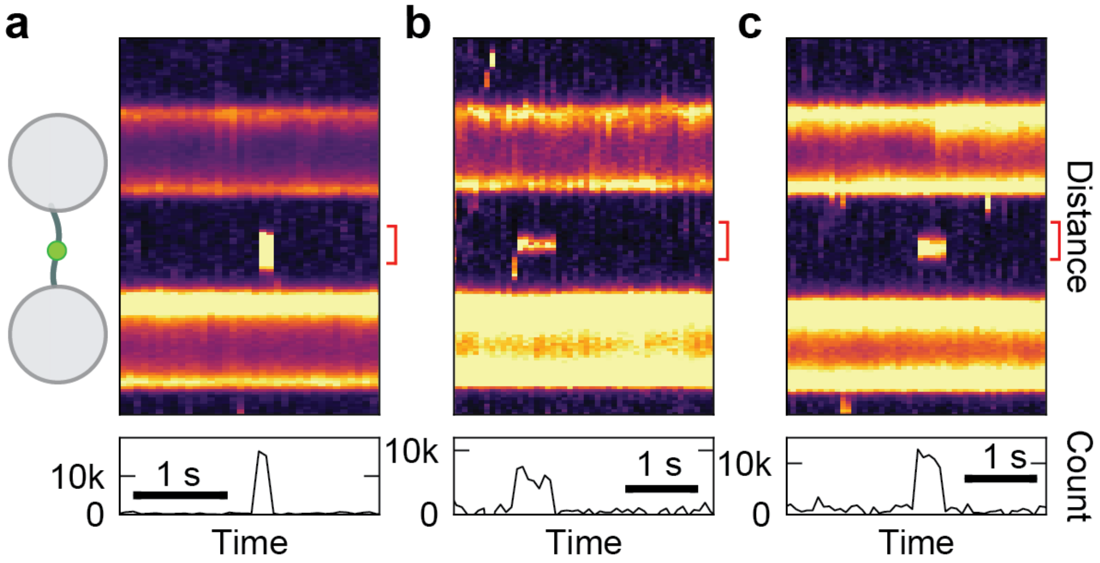

Supplementary Fig. 7: Examples of short binding of trigger factor to MBP. **a**, **b** and **c**, show different traces for different MBP molecules.

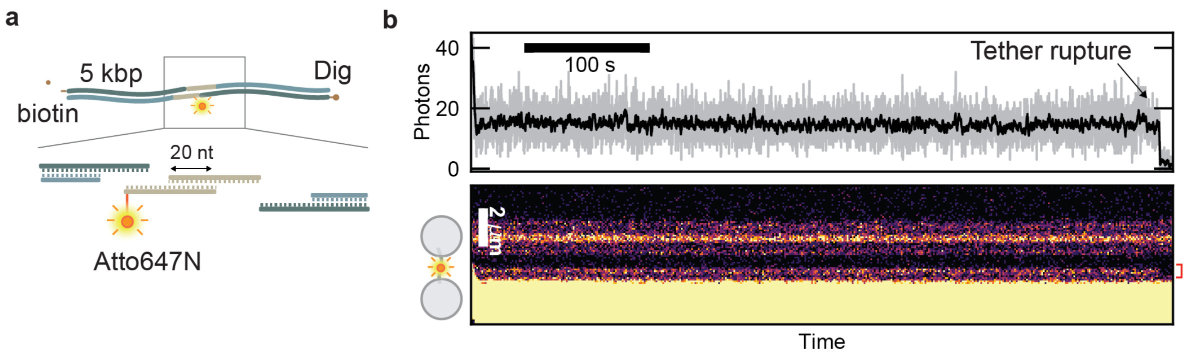

Supplementary Fig. 8: Photobleaching characterization of Atto647N. a, Scheme of the construct used for characterization. Two primers (biomers.net) present an overlapping sequence, which after annealing present identical overhangs that can be hybridized to the longer handles. One of the primers is modified with Atto647N. **b**, Example kymograph of the fluorescence detection for the DNA construct. The bottom bead is coated with the DNA construct, and therefore its parasitic emission is much higher. As a consequence, the position of the dye appears to be closer to the bead (red marker). In all experiments (*N*=6) the tether broke before the dye bleached, with an average tether lifetime of 660±150 s. Observed trigger factor binding was substantially shorter than this lower photobleaching limit.

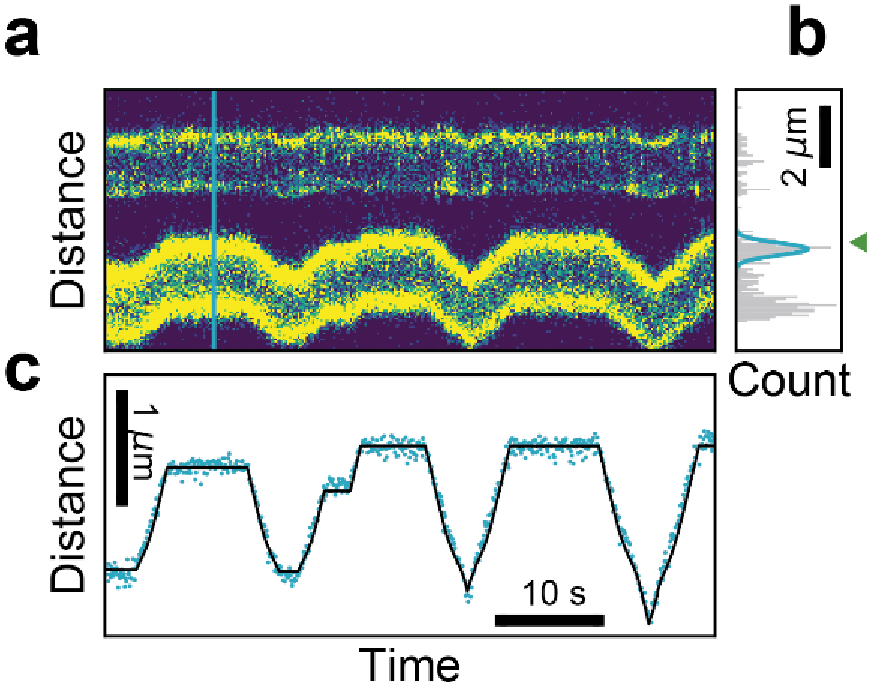

Supplementary Fig. 9: Force spectroscopy and fluorescence signals synchronization. **a**, confocal scanning kymograph of the beads with YPet tethered in between. **b**, profile of a scanning line (blue line in **a**), with a Gaussian fit of the edge of the moving bead in blue. **c**, Synchronization of fluorescence (blue dots, position of bead calculated for each scanning line as shown in **b**) and high-resolution position detector (black line, representing the actual movement of the bead) signals.

Supplementary References

1. Zhou, Z., et al. Genetically encoded short peptide tags for orthogonal protein labeling by Sfp and AcpS phosphopantetheinyl transferases. *ACS chemical biology* **2(5)**: 337-346 (2007).
